# Photoperiodic Influences on Ultradian Rhythms of Male Siberian Hamsters

**DOI:** 10.1371/journal.pone.0041723

**Published:** 2012-07-27

**Authors:** Brian J. Prendergast, Irving Zucker

**Affiliations:** 1 Department of Psychology and Committee on Neurobiology, University of Chicago, Chicago, Illinois, United States of America; 2 Departments of Psychology and Integrative Biology, University of California, Berkeley, California, United States of America; University of Alabama at Birmingham, United States of America

## Abstract

Seasonal changes in mammalian physiology and behavior are proximately controlled by the annual variation in day length. Long summer and short winter day lengths markedly alter the amplitude of endogenous circadian rhythms and may affect ultradian oscillations, but the threshold photoperiods for inducing these changes are not known. We assessed the effects of short and intermediate day lengths and changes in reproductive physiology on circadian and ultradian rhythms of locomotor activity in Siberian hamsters. Males were maintained in a long photoperiod from birth (15 h light/day; 15 L) and transferred in adulthood to 1 of 7 experimental photoperiods ranging from 14 L to 9 L. Decreases in circadian rhythm (CR) robustness, mesor and amplitude were evident in photoperiods ≤14 L, as were delays in the timing of CR acrophase and expansion of nocturnal activity duration. Nocturnal ultradian rhythms (URs) were comparably prevalent in all day lengths, but 15 L markedly inhibited the expression of light-phase URs. The period (τ’), amplitude and complexity of URs increased in day lengths ≤13 L. Among hamsters that failed to undergo gonadal regression in short day lengths (nonresponders), τ’ of the dark-phase UR was longer than in photoresponsive hamsters; in 13 L the incidence and amplitude of light-phase URs were greater in hamsters that did not undergo testicular regression. Day lengths as long as 14 L were sufficient to trigger changes in the waveform of CRs without affecting UR waveform. The transition from a long- to a short-day ultradian phenotype occurred for most UR components at day lengths of 12 L–13 L, thereby establishing different thresholds for CR and UR responses to day length. At the UR-threshold photoperiod of 13 L, differences in gonadal status were largely without effect on most UR parameters.

## Introduction

Ultradian rhythms (URs) have been described for many taxa and persist at multiple levels of biological organization [1], [2]. Prominent functionally significant URs of hormone secretion are well-described in the gonadal, pituitary and adrenal axes of mammals [3]–[5].

In contrast to the abundant research on circadian rhythms, little is known about ultradian rhythms of behavior, with the exception of feeding and locomotor activity of voles and shrews [6]–[8]. and torpor in Siberian hamsters [9]. Seasonal (photoperiod-driven and circannual) changes in the mammalian circadian system have been well-elaborated at formal [9]–[12]. and molecular levels of analysis [13]–[19], but only a few studies have addressed seasonal variations in mammalian URs. In the common vole, a 2 h rhythm in daytime trap catches was detected in winter but not in summer [20]. In reindeer, URs of locomotor activity were significantly shorter in summer than in winter [21]. In Siberian hamsters, the dominant period of the body temperature rhythm also was shorter in long than in short day lengths [9]. The precise day length at which the UR period change occurs, and whether it differs across decreasing short day lengths, has not been investigated. In female Siberian hamsters entrained to long day lengths, multiple quantitative aspects of URs (robustness, mesor, amplitude) differed between the light and dark phases of the photocycle, and the circadian system mediated most of these effects [22]. In addition, an earlier study of Syrian hamsters documented increases in the robustness and amplitude of URs paralleling decreases in the robustness and amplitude of CRs over the course of gestation and lactation [23] along with apparent influences of ovarian hormones on the period and amplitude of URs. Whether changes in entrainment of the circadian system that occur as photoperiods decrease impact the ultradian system is unknown, as is the extent to which testicular hormones affect URs. To address these issues we monitored home cage locomotor activity of adult male Siberian hamsters transferred from a long photoperiod (15 h light/day; 15 L) to one of several day lengths ranging between 14 L to 9 L. This permitted titration of the critical day lengths for induction of photoperiodic responses in the ultradian, circadian and reproductive systems.

## Methods

### Animals and Housing

Siberian hamsters (*Phodopus sungorus*) from a local breeding colony maintained on a light:dark cycle of 15 L (lights off at 18∶00 CST) were housed in polypropylene cages (28×17×12 cm) on wood shaving bedding (Harlan Sani-Chips, Harlan Inc., Indianapolis, IN) with cotton nesting material continuously available. Ambient temperature was 20±0.5°C, and relative humidity 53±2%. Food (Teklad Rodent Diet 8604, Harlan Inc.) and filtered tap water were provided *ad libitum*. In all photoperiods, illuminance was 400–700 lux at cage levls. All procedures conformed to the USDA Guidelines for the Care and Use of Laboratory Animals and were approved by the Institutional Animal Care and Use Committee of the University of Chicago.

### Activity Measurements

Many studies of URs measure foraging or feeding behavior [6], [7], [24], [25]. We measured URs and CRs of spontaneous general locomotor activity– a non-food-specific behavior that correlates highly with daily rhythms of sleep-wakefulness, body temperature, and drinking behavior [26], [27]; in the ultradian domain, locomotor activity correlates positively with feeding rhythms [7]. Locomotor activity studies address qualitative and quantitative aspects of underlying circadian and ultradian timing systems. Hereafter, when referring to “URs” and “CRs” we are referencing locomotor behavior rhythms.

Locomotor activity data were collected in the home cage for a minimum of 10 consecutive days with passive infrared motion detectors (Coral Plus, Visonic, Bloomfield, CT) positioned 22 cm above the cage floor. Motion detectors registered activity when 3 of 27 zones were crossed. Activity triggered closure of an electronic relay recorded by a computer running ClockLab software (Actimetrics, Evanston, IL). Cumulative activity counts were collected at 6 min intervals.

### Photoperiod Manipulations

Male hamsters (n = 95), 60–90 days of age maintained from birth in a 15 L photoperiod were transferred on *week 0* to one of seven day lengths: 9 L (n = 11), 10 L (n = 15), 11 L (n = 15), 12 L (n = 15), 13 L (n = 16), 14 L (n = 14), or 15 L (n = 9). The onset of darkness remained constant (18∶00 CST) in all photoperiods to facilitate entrainment [28]. Home cage activity data were collected between weeks 6 and 12. Reproductive and somatic responses in testis sizes, body mass, and pelage color were monitored at regular intervals. Estimated testis volume (ETV, the product of testis length and the square of testis width) was determined for each hamster on weeks 0, 3, 6, and 12, by measuring the length and width of the left testis under light isoflurane anesthesia through the scrotal skin with analog calipers. ETV is positively correlated with testis mass, circulating testosterone concentrations and spermatogenesis [29], [30]. On weeks 0, 6, and 12, hamsters were weighed (±0.1 g), pelage color was assessed using a scale of 1 to 4 (1 =  agouti, summer fur, 4 =  white, winter fur, as described in [31]), without knowledge of the hamster’s treatment group. For analyses of UR and CR waveforms (see below), sample sizes were increased by incorporating home cage locomotor data from 61 additional hamsters that were subjected to photoperiod manipulations identical to those described above in a study of photoperiod history effects on immune function [32] which was conducted concurrently with the present study.

Hamsters that failed to exhibit gonadal regression (*week 12* ETV≥300) and also did not exhibit a winter pelage (fur score  = 1) in photoperiods ≤12 L were designated nonresponders (NR). Hamsters in 13 L and 14 L with large testes were not categorized as responders or nonresponders, but rather as having developed (ETV≥300) or undeveloped (ETV<300) testes. Unlike hamsters that fail to exhibit gonadal regression in categorically short days (≤12 L), because of aberrant entrainment of the circadian system and failure to expand nocturnal melatonin secretion [33], heterogeneous responses in 13 L and 14 L may be unrelated to circadian anomalies and instead reflect photoperiod history and non-photic effects [34], [35]. Data from hamsters with equivalent circadian entrainment in 13 L and 14 L, but exhibiting divergent reproductive responses, are instructive in determining the impact of reproductive status on URs and CRs.

### Activity Analyses – Data Reduction

#### Ultradian rhythms (URs)

Activity data collected at 6 min intervals were parsed into light-phase only (90–150 data points/24 h) and dark-phase only (90–150 data points/24 h) files. For hamsters in each day length, the number of days and nights sampled was adjusted to approximately equalize the number of data points to 900. Thus, for 15 L hamsters, 10 consecutive nights and 6 consecutive days generated dark-phase and light-phase activity files, each with 900 points; the same arrangement was achieved for 12 L hamsters by sampling 7.5 nights and 7.5 days. Successive days of photophase activity data were concatenated into a single file, as were successive nights of scotophase activity, and separately subjected to Lomb-Scargle periodogram (LSP) and cosinor periodogram analyses, as described in detail elsewhere [22].

#### Circadian rhythms (CRs)

Unparsed files (240 data points/24 h) 10 days in length, were subjected to LSP and cosinor periodogram analyses to extract quantitative CR parameters.

### Activity Analyses – Statistical Analyses

Lomb-Scargle periodogram analyses [36] identified the statistical presence/absence of URs and CRs, and UR *complexity*– the number of significant peaks (distinct periods) in the UR spectrum (range: 0.1–7.9 h; [23]). The level of statistical significance (α) was set to 0.01. Cosinor analyses determined several quantitative measures of behavioral URs (range: 0.1–7.9 h) and CRs (range: 22–26 h): *robustness* (or ‘prominence’, the percent of variance accounted for by the best-fit cosine model, which corresponds to the coefficient of determination R^2^ in regression analyses; [37]); *mesor* (rhythm-adjusted mean value around which the waveform oscillates); *amplitude* (the difference between the peak or trough value and the mesor), expressed as absolute values (activity counts) and relative values referenced to the photophase-specific mesor values); the latter measure incorporates baseline activity levels during each photophase in determining rhythm amplitude. Lastly *acrophase* was computed as the mean time (relative to the onset or offset of light) at which the waveform peaks. The level of statistical significance was set to 0.05.

The LSP detects ultradian periodicities from incomplete evenly-sampled time series, is well-suited for measurement of data binned into separate scotophase/photophase files and optimizes detection of URs by not displaying peaks at multiples of all rhythms detected [38], [39]. Supplemental analyses after completion of LSP analysis [40] were adopted as recommended by Refinetti et al. [37]. The cosinor periodogram [41] is a reliable, preferred curve-fitting tool to quantify rhythm parameters [37].

**Figure 1:**
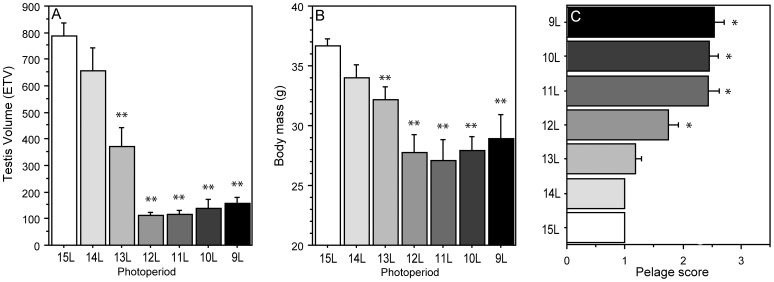
Reproductive, somatic, and pelage responses to decreasing photoperiods. Mean ±SEM (A) estimated testis volume, (B) body mass, and (C) fur score of male Siberian hamsters raised in 15 L and transferred to one of seven experimental photoperiods ranging from 9 L to 15 L *P≤0.05 and **P<0.001 vs. 15 L value.

**Figure 2:**
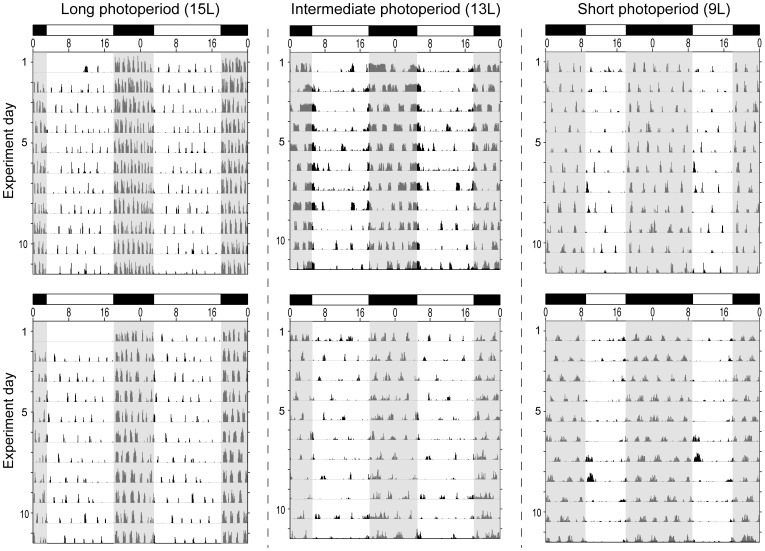
Ultradian and circadian rhythms in locomotor activity. Representative double-plotted home-cage locomotor activity records of two hamsters housed in each photoperiod (15 L,left column), (13 L, center column), and (9 L, right column). Clock time is indicated on the horizontal axis at the top of each actogram, along with light (white) and dark (black) phases of the photocycle. The shaded area overlapping the activity record denotes the daily dark phase.

### General Statistical Analyses

ANOVAs and pairwise comparisons were performed on a computer with Statview 5.0 (SAS Institute, Cary, NC, USA) and LSP and cosinor analyses with software written by R. Refinetti (available at http://www.circadian.org/softwar.html). The proportion of hamsters displaying URs and CRs was evaluated with chi-square tests. The hypothesis being tested was that transfer from 15 L to one of several shorter photoperiods caused a change in UR waveform. To this end, effects of day length on quantitative aspects of URs and CRs, were first examined with ANOVA, and *a priori* planned comparisons were pairwise contrasts between 15 L and each of the 6 shorter day lengths. Planned comparisons were calculated using Fisher’s PLSD tests or unpaired, two-tailed *t* tests. Effects of day length on reproductive and somatic measures were evaluated similarly. Omnibus analyses of pelage scores were performed using the Kruskal-Wallis H test, followed by Mann-Whitney U tests for pairwise comparisons. Differences were considered significant if P≤0.05.

### Multiple Regression and Correlation Analyses

Pearson correlations were calculated to examine the relation between several potential predictor variables (photoperiod, testis size, body mass, and circadian waveform) and two features of the dark-phase UR waveform that respond robustly to decreases in photoperiod: 1) dark-phase UR τ’ and 2) dark-phase UR amplitude. In addition, a multiple regression assessed the relative contributions of photoperiod, reproductive status (ETV) and body mass to dark-phase UR τ’ and amplitude.

To further characterize the manner in which photoperiod affected URs and CRs, non-linear regressions were performed on quantitative parameters of both URs and CRs. Orthogonal polynomial contrast codes were assigned for each of the 7 experimental photoperiods to represent linear, quadratic, and cubic effects. Significant correlations following contrast coding assess whether or not the effect of incrementally-decreasing experimental photoperiods can be characterized by linear, quadratic, or cubic functions, and permit insight into whether photoperiod affects URs and CRs in a similar manner.

**Figure 3:**
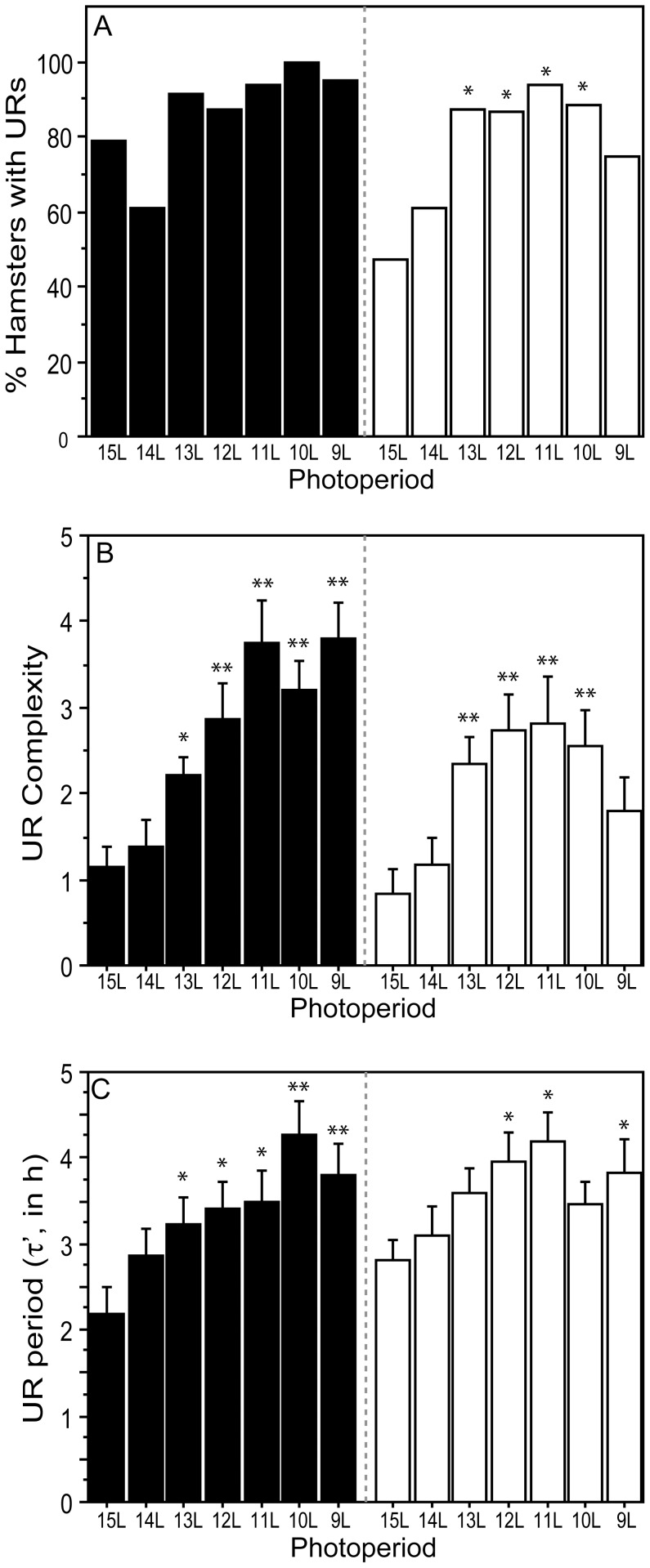
Prevalence, complexity and period of URs in decreasing photoperiods. (A) Percent hamsters exhibiting significant URs during the dark phase (filled/black bars) and during the light phase (open/white bars). (B) Mean ± SEM complexity, and (C) period (τ’) of male Siberian hamsters raised in 15 L and transferred to one of seven experimental photoperiods ranging from 9 L to 15 L *P≤0.05 and **P≤0.005 vs. 15 L value, within photophase.

**Figure 4:**
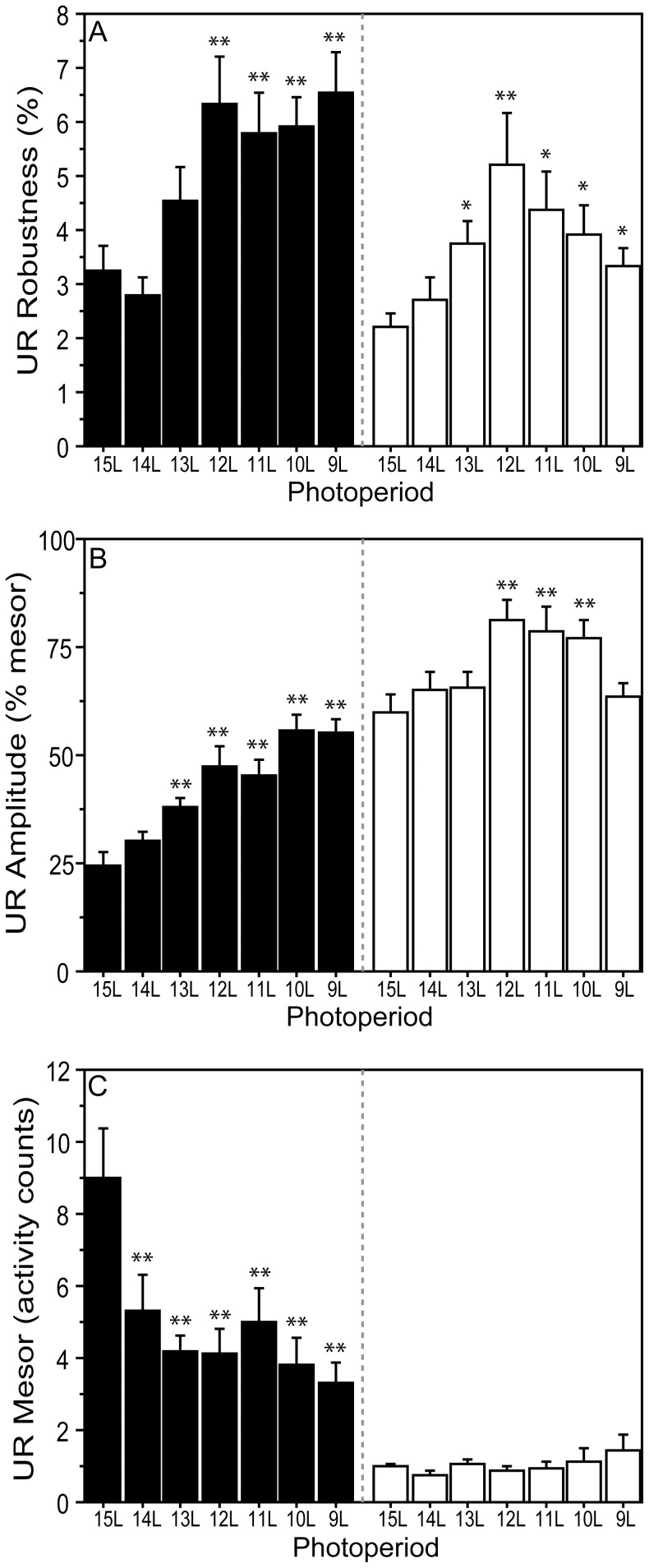
Robustness, amplitude, and mesor of URs in decreasing photoperiods. Mean ± SEM (A) robustness, (B) amplitude, and (C) mesor of the dark phase (filled/black bars) and light phase (open/white bars) ultradian waveforms in 15 L and after transfer to one of seven experimental photoperiods ranging from 9 L to 15 L (indicated along the abscissae). *P≤0.05 and **P≤0.005 vs. 15 L value, within photophase.

**Figure 5:**
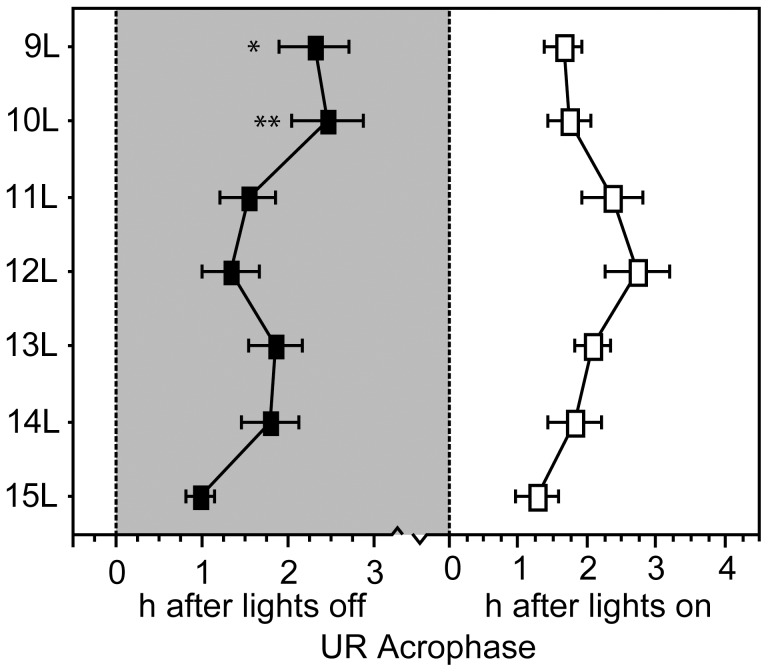
Ultradian rhythm acrophase in decreasing photoperiods. Mean ± SEM acrophase of the ultradian rhythm in 15 L and one of seven experimental photoperiods.*P≤0.05 and **P≤0.005 vs. 15 L value, within photophase.

## Results

### Reproductive and Somatic Responses to Photoperiod

All 11 hamsters in 9 L exhibited gonadal regression. Reproductively nonresponsive hamsters identified in 10 L (n = 4), 11 L (n = 6), and 12 L (n = 5) were removed from the main analysis. In 13 L, 14 L and 15 L, 50%, 21% and 0% of hamsters exhibited gonadal regression (13 L vs. 14 L: χ^2^ = 2.63, P>0.10; 13 L vs. 15 L: χ^2^ = 6.62, P<0.05; 14 L vs. 15 L: χ^2^ = 2.22, P>0.10).

Photoperiod treatments affected testis dimensions (P<0.001; Fig. 1A), body mass (P<0.001; Fig. 1B), and fur color (P<0.001; Fig. 1C) on *week 12*. Among reproductively-responsive hamsters, day lengths ≤13 L resulted in significant gonadal regression (P<0.001 vs. 15 L, all comparisons) and decreases in body mass (P<0.05 vs. 15 L, all comparisons). Pelage moult was observed in day lengths ≤12 L (P<0.05, all comparisons vs. 15 L; cf. [31]).

**Figure 6:**
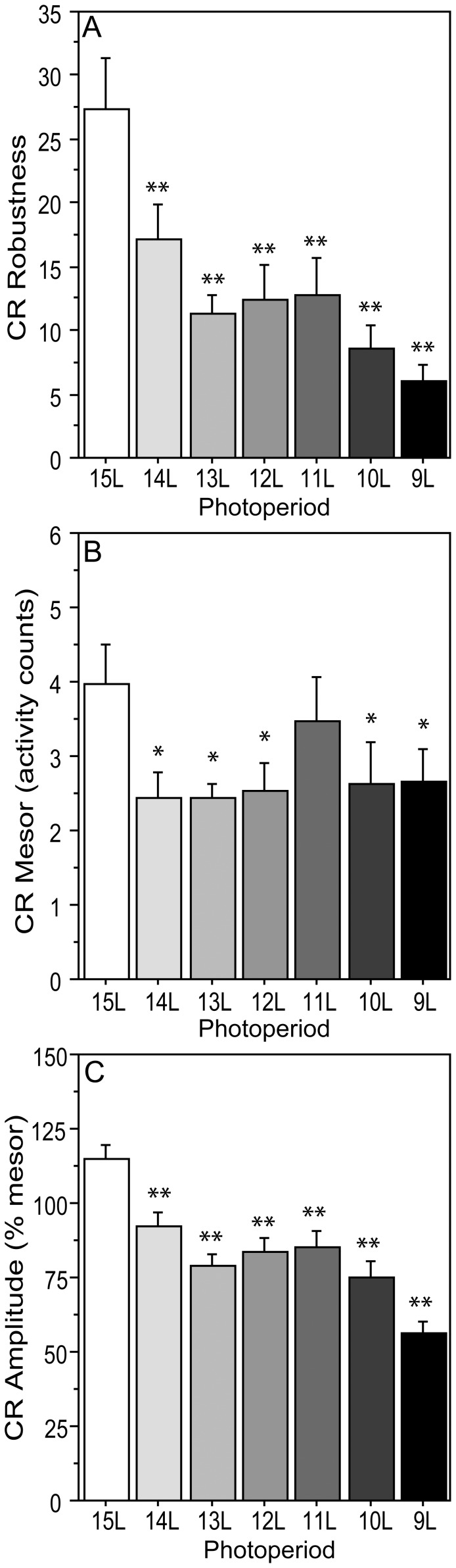
Effects of decreasing photoperiods on robustness, mesor, and amplitude of circadian rhythms. Mean ± SEM (A) robustness, (B) mesor, and (C) amplitude of the circadian waveforms of male Siberian hamsters raised in 15 L and transferred to one of seven experimental photoperiods ranging from 9 L to 15 L (indicated along the abscissae). *P≤0.05 and **P≤0.005 vs. 15 L value.

**Figure 7:**
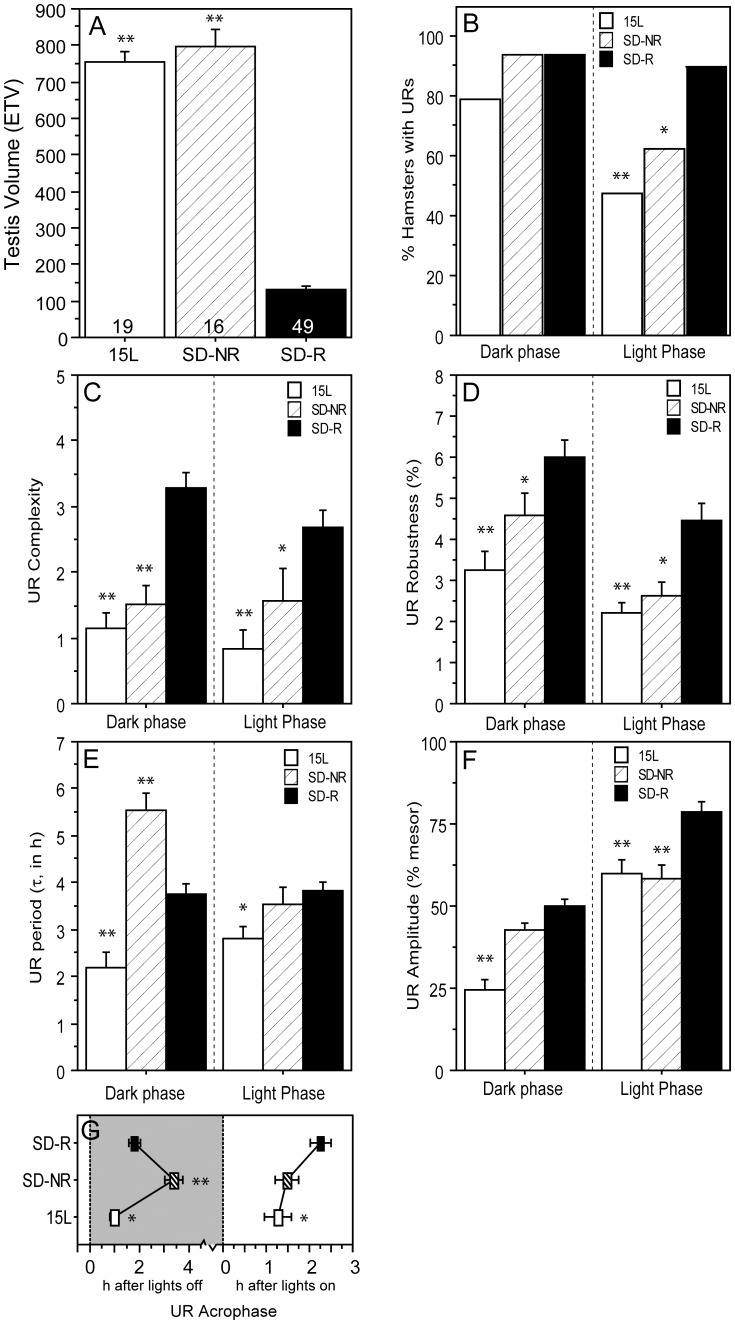
Ultradian rhythms of reproductively non-responsive hamsters. Mean ± SEM (A) testis volumesof 15 L hamsters classified as reproductively responsive (ETV≤300; SD-R) or non-responsive (ETV>300 and fur score  = 1; SD-NR) to short photoperiods ≤12 L. (B) Percent hamsters exhibiting significant URs during the dark phase (left) and light phase (right). Mean ± SEM (C) complexity, (D) robustness, (E) period (τ’), (F) amplitude, and (G) acrophase of the dark phase and light phase ultradian waveforms of 15 L (white bars/symbols), SD-NR (crosshatched bars/symbols) and SD-R (black bars/symbols) hamsters. *P≤0.05 and **P≤0.005 vs. SD-R value.

**Figure 8:**
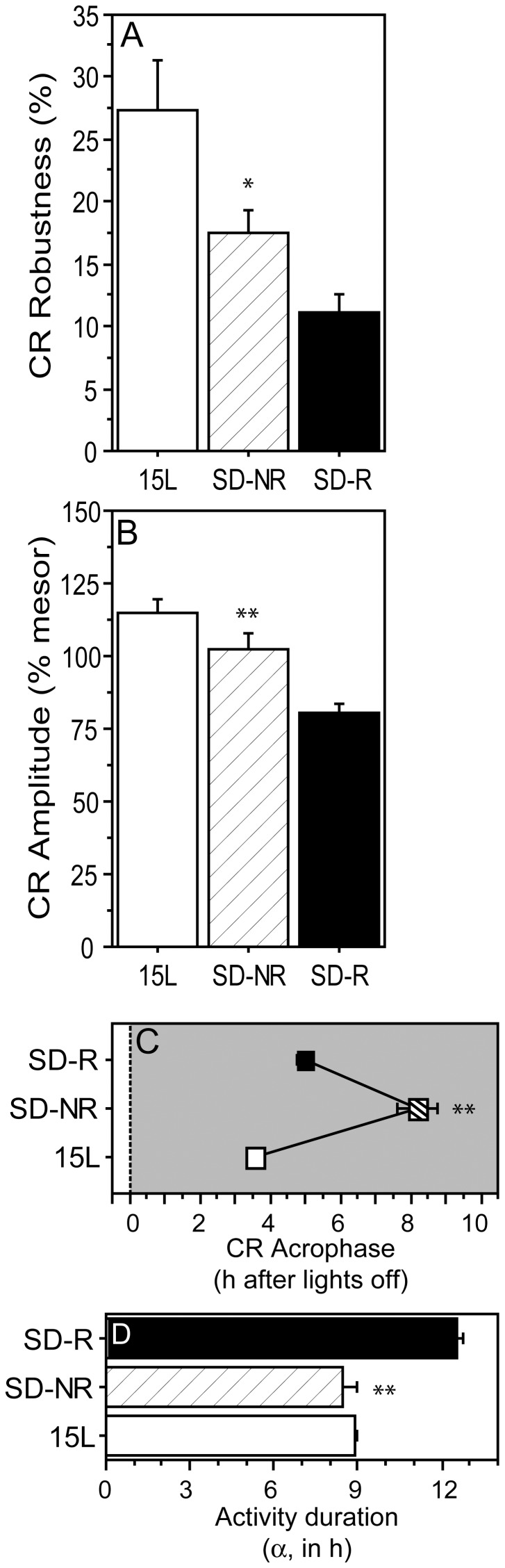
Circadian rhythms of reproductively non-responsive hamsters. Mean ± SEM (A) robustness, (B) amplitude, and (C) acrophase of the circadian waveforms of 15 L (white bars/symbols), SD-NR (crosshatched bars/symbols) and SD-R (black bars/symbols) hamsters. (D) Mean ± SEM duration of nocturnal locomotor activity. *P≤0.05 and **P≤0.005 vs. SD-R value.

### Ultradian Rhythms

For the analysis of locomotor activity data, sample sizes were increased by incorporating data from 61 additional hamsters (9 L: n = 9, 10 L: n = 9, 11 L: n = 8, 12 L: n = 8, 13 L: n = 8, 14 L: n = 9, 15 L: n = 10), treated concurrently and in an identical fashion in a study of immune function (Prendergast and Pyter, 2009). None of the data in the present study were included in the prior report, which did not investigate URs, their relation to CRs, or the several circadian components affected by day length considered herein.

#### Dark-phase URs

Most hamsters exhibited dark-phase URs (Fig. 2; Fig. 3A). UR incidence ranged from 60–100%, but was not influenced by day length (P>0.10, all comparisons). UR complexity (Fig. 3B) and period (Fig. 3C, P<0.005) increased in day lengths ≤13 L; UR robustness increased in photoperiods ≤12 L (Fig. 4A, P<0.001). UR amplitude increased in day lengths ≤13 L (Fig. 4B), and mesor activity levels were significantly lower in all day lengths shorter than 15 L (Fig. 4C, P<0.001). Short day lengths shifted the acrophase of dark-phase URs to later times in 9 L and 10 L, as compared to15 L (Fig. 5; P<0.05 both comparisons).

#### Light-phase URs

The proportion of hamsters displaying light-phase URs was greater in photoperiods ≤13 L compared to 15 L (Fig. 2; Fig. 3A, 13 L through 10 L: P<0.05, all comparisons; 9 L: P = 0.08). URs were more prevalent in the dark- than the light-phase in 15 L (P<0.05), but not in other day lengths (P>0.05, all comparisons). Most day lengths ≤13 L increased UR complexity (Fig. 3B, P<0.001). A main effect of day length on light-phase UR period (τ’) fell short of statistical significance (Fig. 3C, P = 0.07), but τ’ was significantly longer in 12 L, 11 L and 9 L than in 15 L (P<0.05, all comparisons). UR robustness was greater in all day lengths ≤13 L (Fig. 4A, P<0.005). UR amplitude (Fig. 4B, P<0.005) was significantly greater in 12 L, 11 L, and 10 L than in 15 L (P<0.005, all comparisons). Mesor values were not affected by changes in day length (Fig. 4C) and no main effect of photoperiod was evident on the timing of acrophases (Fig. 5, P>0.15).

**Figure 9:**
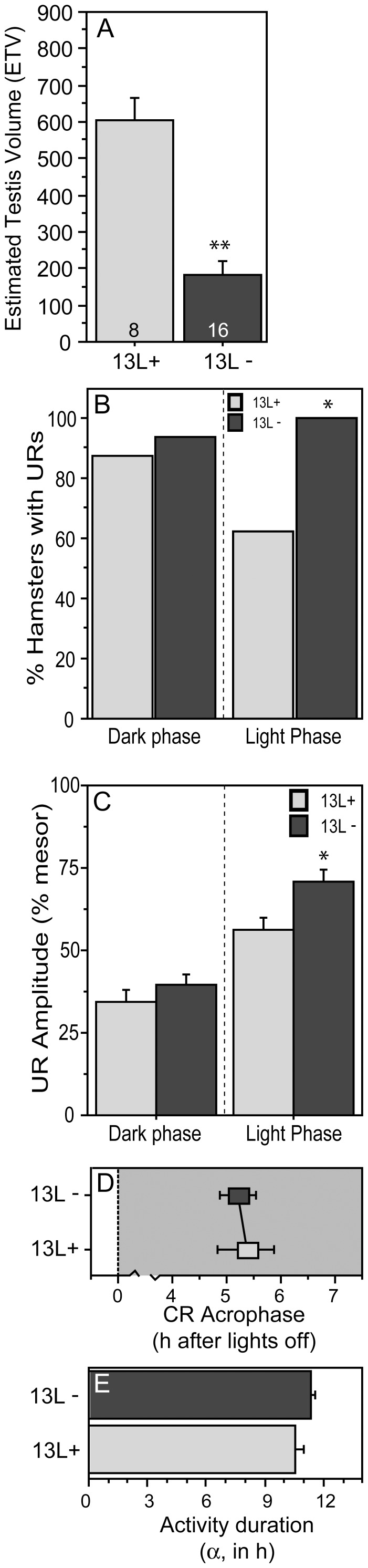
Ultradian rhythms of hamsters exhibiting divergent reproductive responses to the intermediate-duration ( 13 L**) photoperiod.** Mean ± SEM (A) testis volumes of hamsters that exhibited gonadal regression (ETV≤300; 13 L−; black bars/symbols) or maintained developed gonads (ETV>300; 13 L+; grey bars/symbols) in a 13 L photoperiod. (B) Percent of 13 L+ and 13 L− hamsters exhibiting significant URs during the dark phase (left) and light phase (right). (C) Mean ± SEM amplitude of the ultradian waveform of 13 L+ and 13 L− hamsters. (D) Acrophase of the circadian waveform and (E) duration of nocturnal locomotor activity (α) of 13 L+ and 13 L− hamsters. *P≤0.05 and **P≤0.005 vs. 13 L+ value, within photophase.

### Circadian Rhythms

Circadian organization was markedly altered by decreases in day length (Fig. 6). CR robustness was greater in 15 L than in all other photoperiods (Fig. 6A, P<0.005, all comparisons), and did not differ among hamsters in day lengths from 9 L through 13 L. Mesor values were greater in 15 L than in all in other day lengths (Fig. 6B, P<0.05, all comparisons), except 11 L (P>0.40). CR amplitude was lower in 9 L than in all other day lengths (Fig. 6C, P<0.001, all comparisons), and higher in 15 L than in all other photoperiods (P<0.01, all comparisons). Circadian acrophases occurred progressively later in shorter photoperiods (P<0.001) and the duration of the nocturnal active phase (α) increased incrementally from 8.9±0.06 h in 15 L to 11.4±0.19 h in 9 L (mean ± sem; P<0.001).

### URs and CRs in Nonresponder (NR) Hamsters

URs were compared between short-day (9 L through 12 L, inclusive) hamsters that underwent gonadal regression (responders, SD-R) and those that maintained large testes (nonresponders, SD-NR). Because there were no quantitative differences in URs of SD-NR hamsters in 10 L, 11 L and 12 L (n = 4–6 per group), a single SD-NR group was constituted for purposes of analysis (n = 16). UR waveforms of SD-R and SD-NR hamsters were compared to those of 15 L hamsters (n = 19).

#### Dark-phase URs

UR prevalence was high (79–90%) and did not differ significantly between SD-R and SD-NR hamsters (P>0.80; Fig. 7B), but UR complexity (Fig. 7C, P<0.001) and UR robustness (Fig. 7D, P = 0.05) were significantly lower in SD-NR relative to SD-R hamsters; on these measures, SD-NR hamsters were comparable to 15 L hamsters. Dark-phase UR period was substantially longer in SD-NR hamsters than in SD-R hamsters (Fig. 7E, P<0.001). Mesor and amplitude (Fig. 7F) values were comparable in SD-NR and SD-R hamsters (P>0.05; all comparisons), but acrophases of SD-NR hamsters occurred significantly later than those of SD-R hamsters (Fig. 7G, P<0.001).

#### Light-phase URs

URs were less prevalent in SD-NR than in SD-R hamsters (Fig. 7B, P = 0.01). UR complexity (P<0.05) and robustness (P = 0.01) were lower in SD-NR than SD-R hamsters (Fig. 7C,D), but τ’ did not differ between these groups (Fig. 7E, P>0.30). Mesor values were low and comparable in SD-NR and SD-R hamsters (P>0.70), but UR amplitude was lower in SD-NR than SD-R hamsters (Fig. 7F, P<0.001). Acrophases did not differ significantly between SD-R and SD-NR hamsters (Fig. 7G).

#### Circadian rhythms

CR robustness (Fig. 8A, P<0.05) and amplitude (Fig. 8B, P<0.005) were significantly greater in SD-NR than SD-R hamsters; CR acrophases occurred >3 h later in SD-NR hamsters (Fig. 8C, P<0.001). Duration of the active phase was substantially shorter in SD-NR hamsters (Fig. 8D, P<0.001; cf. [42], [43]).

### CRs and URs in the Intermediate-duration Photoperiod

Divergent reproductive responses were evident in 13 L [34], [44]: 16 hamsters exhibited gonadal regression (13 L− group), whereas 8 retained large testes (13 L+ group) (Fig. 9A, P<0.001).

#### Ultradian Rhythms

Dark-phase URs were evident in 94% of 13 L− and 88% of 13 L+ hamsters (Fig. 9B, P>0.60). In contrast, light phase URs were present in 100% of 13 L− but in only 63% of 13 L+ hamsters (Fig. 9B, P<0.05).

Quantitative aspects of dark-phase URs (complexity, τ’, robustness, mesor, amplitude, acrophase) did not differ between 13 L+ and 13 L− hamsters. Light-phase UR complexity, τ’, robustness, mesor and acrophase also were similar in 13 L + and 13 L− hamsters (P>0.10, all comparisons), but amplitude of the light-phase UR was greater in 13 L− than in 13 L+ hamsters (Fig. 9C, P<0.05).

#### Circadian Rhythms

CR acrophase and circadian α were comparable in 13 L+ and 13 L− hamsters (Fig. 9D, 9E; P>0.40, both comparisons). CR robustness, mesor and amplitude were also indistinguishable between 13 L+ and 13 L− groups (P>0.10, all comparisons).

### Simple and Multiple Regression Analyses

Day length (R = −0.36; P<0.001) and testis size (R = −0.21; P<0.01) were negatively correlated with dark-phase UR τ’, whereas body mass did not predict τ’ (P>0.50; Table 1). Robustness of the circadian waveform (R = 0.17; P<0.05), mesor activity levels (R = 0.27, P<0.01) and CR acrophase (R = 0.29, P<0.001) were each positively correlated with dark-phase UR τ’. Significant negative correlations were observed between dark-phase UR amplitude and photoperiod (R = −0.62), testis size (R = −0.57) and body mass (R = −0.57; P<0.001 all correlations). In the circadian waveform, CR robustness (R = −0.64), mesor (R = −0.47) and amplitude (R = −0.44) were negatively correlated with UR amplitude (P<0.001, all correlations), and CR acrophase (R = 0.19, P<0.05) and nocturnal α (R = 0.47; P<0.001) were positive predictors of dark-phase UR amplitude (Table 1).

**Table 1:** Least squares regression models of single (top) and multiple (bottom) predictor variables on dark-phase UR τ’ and dark-phase UR amplitude after photoperiod manipulations.

*Simple linear regressions*										
	Pearson correlations (R)									
Variable	mean			SD		UR τ’			UR amplitude	
photoperiod†	–			–		−.364***			−.620***	
body mass	31.0			5.7		−.054			−.567***	
testis size	338.3			297.4		−.214**			−.570***	
circadian α (h)	11.4			2.21		.117			.468***	
CR robustness (%)	13.			12.5		.174			−.637***	
CR mesor (counts)	2.83			1.94		.272**			−.465***	
CR amplitude (% mesor)	83.7			26.3		.009			−.436***	
CR acrophase (radians)	1.34			0.52		.293***			.187*	
***Least-squares multiple regression model***										
		**Dark-phase UR τ’**					**Dark-phase UR amplitude**			
**Predictor variable**		**b**	**±SE**		**β**		**b**	**±SE**		**β**
photoperiod†		−.349***	.091		−.458		−.034***	.008		−.401
body mass		.050	.029		.189		−.009***	.003		−.302
testis size (ETV)		<.001	<.001		−.005		<.001	<.001		−.081

* P<0.05, **P<0.01, ***P<0.001.

† coded as 1 = 9 L, 2 = 10 L, 3 = 11 L, 4 = 12 L, 5 = 13 L, 6 = 14 L, 7 = 15.

To examine if effects of photoperiod on URs are mediated by concurrent changes in reproductive condition or body mass, a multiple regression model constructed of 3 components (photoperiod, week 12 body mass and week 12 ETV) was constructed. This model significantly predicted dark-phase UR τ’ (R^2^ = 0.22, F3,130 = 7.94, P<0.001; standard error of the estimate  = 1.42 h) and dark-phase UR amplitude (R^2^ = 0.47, F3,129 = 36.6, P<0.001; standard error  = 0.129; Table 1). The effect of photoperiod on both τ’ and UR amplitude was significant (P<0.001) in this model (Table 1). The effects of testis size on τ’ and on UR amplitude, which were present as zero-order effects in the simple linear regression, were not significant when photoperiod was included in the model. However, a significant negative effect of body mass on UR amplitude was obtained in the multiple regression model (partial correlation coefficient  = −0.009, P<0.001; Table 1).

### Linear, Quadratic and Cubic Contrasts

Linear regression analyses on quantitative parameters of dark-phase URs revealed significant simple (linear) effects of photoperiod on all measures of the waveform (Table 2); higher-order (quadratic, cubic) contrasts were not significant with the exception of dark-phase UR mesor. Linear effects were significant for most measures of the light-phase UR waveform, except UR mesor and UR acrophase. Higher-order quadratic contrasts were significant for all measures of the light-phase UR waveform except UR mesor, but cubic contrasts were non-significant. Lastly, analyses of the CR waveform revealed significant simple linear contrasts for robustness, amplitude and acrophase. Quadratic effects of photoperiod change were largely absent, but higher-order cubic contrasts were significant for all measures of the CR waveform (Table 2).

**Table 2:** UR and CR responses to photoperiod: linear, quadratic, and cubic contrasts.

Variable	|*t*| linear	|*t*| quadratic	|*t*| cubic
*Ultradian rhythms*			
Dark-phase			
complexity	7.75***	1.48	1.49
τ’	4.19***	1.29	0.42
robustness	5.47***	1.22	1.29
Mesor	4.17***	2.35*	2.02*
amplitude	8.63***	1.47	1.21
acrophase	2.42*	0.19	0.86
Light-phase			
complexity	3.19**	3.85***	1.28
τ’	2.72**	1.96*	0.51
robustness	2.47*	3.49***	0.86
Mesor	1.62	1.06	0.19
amplitude	2.23*	3.54***	2.33*
acrophase	0.85	2.78**	0.12
*Circadian rhythms*			
robustness	5.97***	2.10*	2.38*
Mesor	0.93	1.39	2.24*
amplitude	7.90***	0.84	3.29***
acrophase	5.29***	0.98	3.07**

Symbols indicate significance at *P<0.05, **P<0.01, ***P<0.001.

## Discussion

Earlier reports suggested that ultradian body temperature rhythms of Siberian hamsters are substantially longer in a short (8 L) than a long (16 L) day length [9]. The period estimates of 1.6 h in long days and 3.7 h in short days were based on 24 h analyses that encompassed both the light and dark phases [9]. The present investigation revealed substantial effects of photoperiod on multiple components of the ultradian waveform, which in many cases differed in the active (dark) versus the inactive (light) phases, suggesting that behavioral analyses are most informative if restricted to a given photophase. This approach established period lengthening of the ultradian locomotor rhythm in both photophases as day lengths decreased from 15 h to ≤13 h. The expression of dark phase locomotor URs was not affected by variations in day length, but hamsters were significantly more likely to express light phase URs in short than in long days, establishing photophase-specific seasonal variation in ultradian organization.

The amplitude of URs was enhanced in both the light and dark phases after the transition from a long to one of several shorter day lengths. The critical day length for these transitions ranges from 12 to 14 h for the several ultradian components. The latency for instatement of the short-day ultradian phenotype is presently unspecified.

Day length induced parallel changes in circadian organization. Circadian amplitude, and robustness were greater in 15 L than in all short day lengths ≤13 L. The acrophase of the circadian locomotor rhythm occurred later in short days, and nocturnal α expanded as day length decreased, as previously noted [28]. A functional circadian system is not required for the generation of URs. URs persist in Siberian hamsters rendered arrhythmic after a regimen of disruptive phase shifts [22], [45]; in rats, Syrian hamsters and common voles, URs in behavior and physiology also persist after surgical ablation of the suprachiasmatic nucleus[46]–[49], but see [50]. Although URs are not dependent on a functional circadian system, circadian activity exerts modest influences on URs. An increase in the number of significant URs is positively correlated with the power of Syrian hamster free-running circadian rhythms [48] and hamsters bearing the *tau* mutation have shorter UR periods in feeding [51] and locomotor activity [52] relative to wild-type hamsters. And, absent circadian organization, day-night rhythms in quantitative features of the UR waveform (robustness, mesor activity levels, amplitude) are abolished [22]. In the present study, across all day lengths, decreases in the amplitude of CRs were significantly correlated with increases in the amplitude of URs. A similar relation was recently observed in Syrian hamster dams–beginning early in gestation and persisting through weaning, CR amplitude and robustness were significantly diminished, whereas UR complexity, robustness and amplitude were markedly increased [23]. Decrements in the amplitude of the circadian system, whether a consequence of short photoperiods or gestation, may be a prerequisite for emergence of ultradian power.

Some Siberian hamsters fail to undergo testicular regression in short day lengths (nonresponders; reviewed 33, 53). The dark phase τ’was substantially longer (5.5 h) in males that sustained large testes in short day lengths (10 L–12 L) than in those whose testes were regressed (3.8 h); such differences were absent in the light phase, emphasizing the importance of photophase-specific analyses. At present the increase in τ’ in nonresponder hamsters appears paradoxical. In Siberian hamsters, blood testosterone concentrations of SD responders are reduced to about 10% of LD values (e.g., [54]), but this decrease is unlikely to account for the above τ’ differences; nonresponder τ’ s are much longer than those of hamsters housed in long day lengths (15 L), yet both groups have equally large testes and presumably generate comparable blood androgen concentrations. The duration of nightly melatonin secretion is substantially shorter in nonresponder than responder hamsters [55]; if the nocturnal melatonin signal influences τ’, as suggested by Heldmaier et al. [9], then one would anticipate that τ’ would be shorter in nonresponders than responders and comparable to that of long day (15 L) hamsters, with comparably short duration nocturnal melatonin secretion, but this outcome was not observed. Modified circadian organization in nonresponders, including the delayed acrophase (Fig. 7C), and altered phase response curves to light pulses [42], rather than changes in hormone secretion, may be causally related to changes in the ultradian period (cf. [7], [23]). On the other hand, the robustness and amplitude of CRs, which are greater in nonresponder than responder hamsters may reflect decreased androgen secretion. In Syrian hamsters [56], [57] the integrity of wheel running circadian rhythms decreases in short day lengths.

Gonadal steroid modulation of seasonal changes in URs remains to be elaborated. In Syrian hamsters ovarian hormones profoundly influence ultradian locomotor organization [23] hamsters with elevated estradiol and progesterone during pregnancy exhibit increases in complexity, robustness, and amplitude of dark-phase URs. In Siberian hamsters, SD-induced changes in circadian and gonadal function are usually tightly linked [42], [43]. The increase in dark-phase ultradian τ’, complexity, robustness and amplitude in shorter day lengths is correlated with decreased gonadal androgen and gonadotrophin secretion in shorter days [54], [58], but it is also correlated with changes in the entrainment state of the circadian system. Manipulations of gonadal steroids in hamsters maintained in a fixed LD photoperiod are required to assess the relative contributions of circadian and gonadal responses to seasonal changes in ultradian structure.

Divergent responses of hamsters to the 13 L photoperiod provide additional insight into seasonal modulation of ultradian structure by the circadian system and gonadal steroids. Circadian entrainment (acrophase, α) and power (robustness, mesor, amplitude) were comparable in 13 L+ and 13 L− hamsters, but these groups, by definition, exhibited profound differences in gonadal function. Nevertheless, gonadal phenotype did not affect dark-phase URs in 13 L. Only a modest increase in light-phase UR amplitude was evident in 13 L hamsters. The absence of any systematic effect of reproductive phenotype in 13 L hamsters suggests that photoperiodic changes in quantitative aspects of URs occur via mechanisms largely independent of concurrent changes in gonadal hormone secretion.

Threshold photoperiods for initiating the transition to the short-day phenotype differed for the circadian and ultradian systems. Photoperiods as long as 14 L were sufficient to trigger decreases in CR robustness, mesor, amplitude, acrophase and α. In contrast, increases in dark-phase UR complexity, period, and amplitude required photoperiods ≤13 L; increases in robustness occurred at 12 L, and delays in acrophase occurred at 10 L. This suggests that significant decreases in the amplitude or robustness of circadian pacemaker output are not sufficient to induce SD-like enhancements in ultradian rhythm amplitude. Photoperiod-driven changes in CR amplitude may interact with putative gonadal hormone effects to influence the UR waveform.

Quantitative comparison of circadian and ultradian responses to day length (Table 2) with regression analyses revealed significant linear effects of decreasing photoperiod on all quantitative aspects of dark-phase URs except mesor activity. Higher-order effects of photoperiod were absent on dark-phase URs, indicating that incremental decreases in photoperiod induce proportional effects on dark-phase UR complexity, τ’, robustness, amplitude, and acrophase (cf. Fig. 3B, 4B, 5). In contrast, higher-order effects of photoperiod were evident on light-phase URs. These were primarily quadratic effects, indicating that as day lengths decrease, there is a non-linear acceleration of the impact of photoperiod change on light-phase URs (cf. Fig. 3B, 4A, 5). Higher-order responses to day length were also evident in all measures of CRs (robustness, mesor, amplitude, acrophase), but these were uniformly *cubic* in nature, indicating that circadian responses to decreasing day lengths are best characterized by a step function. For example, decreases in CR amplitude occur abruptly upon transfer from 15 L to 14 L, followed by a plateau from 13 L through 10 L, followed by further decreases in 9 L (cf. Fig. 6C). The mechanisms responsible for these asymmetries between circadian and ultradian responses to photoperiod remain unspecified.

Correlation and multiple regression analyses examined the relation between aspects of the dark-phase UR waveform that exhibit robust responses to decreasing day length (τ’ and amplitude) and various potential predictors. Photoperiod and testis size were each negatively and significantly correlated with increases in τ’; similar effects were also evident on dark-phase UR amplitude; in addition, week 12 body mass was a significant negative predictor of UR amplitude (Table 1). When photoperiod is included in the model, effects of testis size on τ’ and UR amplitude disappear, suggesting that any effects of reproductive condition on UR τ’ and amplitude may be mediated via photoperiod effects on the reproductive system. However, the partial correlation between body mass and UR amplitude persists in the multiple regression model, indicating a contribution of body mass to UR amplitude even when strong effects of photoperiod are taken into account. The magnitude of such photoperiod-independent effects of body mass are modest: according to this model, a 1 g decrease in body mass would be expected to yield an increase in UR amplitude of ∼1%. The prediction based on these data is that heavier hamsters would be predisposed to lower-amplitude URs, independent of photoperiod.

Table 1 also summarizes results of a simple regression analyses of the relative contributions of several components of the circadian waveform to dark-phase τ’ and amplitude. Small positive correlations were obtained between CR mesor and CR acrophase, and τ’. More robust simple effects were evident on UR amplitude. Nocturnal α was a strong positive predictor of UR amplitude; shorter αs were linked to higher-amplitude dark-phase URs. The close temporal relation between nocturnal melatonin secretion and the duration of α [11], [42], [59] suggests that photoperiod-mediated changes in melatonin secretion, independent of changes in the circadian waveform, influence this aspect of dark-phase URs. CR robustness, mesor and amplitude were each large negative predictors of UR amplitude, indicating that hamsters with robust, high-amplitude CR waveforms and high activity levels tended to have low-amplitude URs.

The functional significance of photoperiodic changes in behavioral URs remains a matter of conjecture. The short-day ultradian phenotype can be induced by maintaining long-day hamsters in low ambient temperatures [9], suggesting that the longer period of the UR in short days, which in nature coincides with lower environmental temperatures, imposes longer rest periods that conserve energy [9].

In summary the present work titrated effects of photoperiod on quantitative aspects of CRs and URs. A significant negative relation exists between amplitude of the circadian and ultradian systems in this species, but SD-like changes in several quantitative aspects of the CR waveform were evident at photoperiods inadequate to trigger changes in the UR waveform. Future studies will be necessary to disentangle the respective contributions of gonadal hormones from direct or indirect contributions of the circadian system to the genesis of short-day induced changes in the UR waveform. However, at the threshold photoperiod of 13 L, categorical differences in reproductive condition were without effect on most UR measures, suggesting that changes in gonadal hormone secretion are not sufficient mediators of seasonal changes in URs.
